# Evaluation of flow changes after telescopic stenting of a giant fusiform aneurysm of the vertebrobasilar junction

**DOI:** 10.1186/s12938-019-0699-1

**Published:** 2019-07-24

**Authors:** Sergey Sindeev, Jan Stephan Kirschke, Sascha Prothmann, Sergey Frolov, Dieter Liepsch, Philipp Berg, Claus Zimmer, Benjamin Friedrich

**Affiliations:** 1 Department of Biomedical Engineering, Tambov State Technical University, Tambov, Russia; 2 Department of Diagnostic and Interventional Neuroradiology, Klinikum Rechts der Isar, Technical University of Munich, Ismaninger Strasse 22, 81675 Munich, Germany; 3 Department of Neuroradiology, Helios Klinikum München West, Munich, Germany; 4 Department of Building Services Engineering, Munich University of Applied Sciences, Munich, Germany; 5 Research Campus STIMULATE, University of Magdeburg, Magdeburg, Germany

**Keywords:** Posterior circulation, Intracranial aneurysm, Fusiform aneurysm, Vertebrobasilar junction, Flow-diverter, Computational fluid dynamics

## Abstract

**Background:**

The use of flow-diverters for non-saccular cerebral posterior circulation aneurysms requires complex deployment techniques and is associated with high mortality and morbidity. Therefore, further studies are required to clarify the effect of stenting on post-treatment hemodynamics in such aneurysms. In this study, we evaluated flow alterations in a treated giant fusiform aneurysm of the vertebrobasilar junction and correlated them with the clinical outcome.

**Methods:**

A patient-specific aneurysm model was acquired by rotational angiography, and three SILK flow-diverters (4.5 × 40, 5 × 40 and 5.5 × 40 mm) were virtually deployed in series along the basilar and right vertebral arteries. Image-based blood flow simulations before and after the treatment were performed under realistic pulsatile flow conditions. The flow reduction, velocity and wall shear stress (WSS) distribution, streamlines and WSS-derived parameters were evaluated before and after the treatment.

**Results:**

The computed velocity streamlines showed substantial alterations of the flow pattern in the aneurysm and successful redirection of blood flow along the series of flow-diverters with no flow through the overlapping stents. The obtained flow reduction of 86% was sufficient to create thrombogenic flow conditions. Moreover, a 6.2-fold increase in relative residence time and a decrease by 87% of time-averaged WSS contributed to a successful treatment outcome observed during the follow-up.

**Conclusions:**

We found a correlation between the numerically predicted flow alterations and the available treatment outcome. This shows the potential of image-based simulations to be used in clinical practice for treatment planning and estimation of possible risk factors associated with a complex stent deployment in fusiform aneurysms of the posterior circulation.
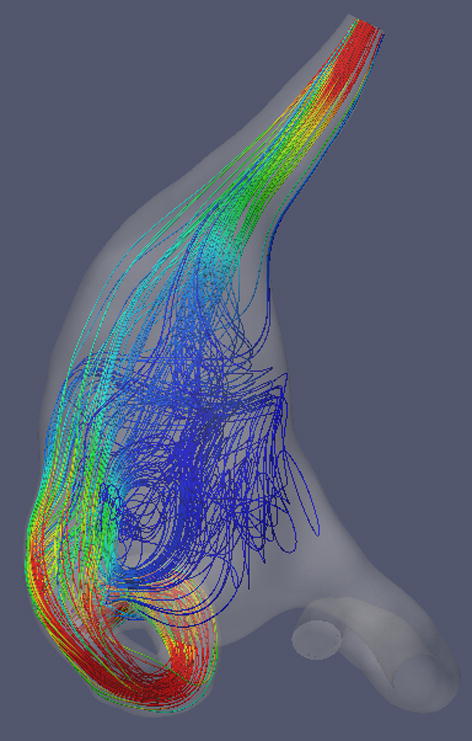

## Background

A fusiform aneurysm is a circumferential dilatation of the vessel with diameter more than 1.5 of the nominal and a non-detectable neck, which occurs more often in the posterior (40% of aneurysms) than in the anterior circulation (only 15%) [1]. Moreover, the rupture risk of cerebral posterior circulation aneurysms is approximately four times higher compared to aneurysms in the anterior circulation (1.8% vs 0.49%, respectively) with complete occlusion rate after endovascular treatment of only 71.2% [1, 2]. The most common site of posterior fusiform aneurysms is the vertebrobasilar junction. Despite the fact that fusiform aneurysms of the vertebrobasilar arterial system are relatively rare, their treatment is associated with low occlusion rate and high risk of major post-treatment complications compared to saccular aneurysms [3, 4].

Several studies have shown a poor outcome for both surgical and endovascular treatment options of posterior circulation aneurysms [5, 6]. In the majority of cases, only endovascular coil occlusion of one of the vertebral arteries is not sufficient to reduce the aneurysm growth; therefore, flow-diverter stents could be used as an adjunctive device [7]. Initially, flow-diverter stents were employed for the treatment of saccular aneurysms of the anterior circulation; however, due to high efficiency, their application has been extended to fusiform aneurysms and aneurysms of the posterior circulation [8–10].

A flow-diverter is a highly braided stent aimed to recover a natural blood flow in the cerebral artery and initiate aneurysm thrombosis [11]. Since the appearance in clinical practice in 2007, flow-diverter stents became a useful tool for treating complex, wide-necked aneurysms, which could not be treated with traditional clinical methods such as coiling and clipping [12, 13]. Several multicenter long-term studies reported a high occlusion rate and low risk of complications during the follow-up, although for some specific cases, especially for giant fusiform aneurysms, the complication rate is much higher [14, 15]. Li et al. studied ten fusiform aneurysms of vertebral arteries treated only by flow-diversion, which included three that were unsuccessful [16]. Additionally, the use of flow-diverter stents for non-saccular posterior circulation aneurysms requires complex deployment techniques and is associated with high mortality and morbidity [17]. Therefore, a relatively low occlusion rate and high risk of post-treatment complications make the treatment planning quite challenging. Also, it should be noted that the causes of complications and delayed aneurysm occlusion are still unclear and further studies are required [17, 18]. Thus, studying a flow-diversion effect particularly in the posterior circulation aneurysms is of interest, since in such aneurysms specific flow conditions could exist especially in cases with complex stenting. These flow conditions influence the treatment outcome and probably could explain why flow-diversion in the posterior circulation aneurysms is not as effective as in the anterior circulation aneurysms.

In the present study, we evaluated flow alterations in a giant fusiform aneurysm of the vertebrobasilar junction treated with three flow-diverter stents deployed in series using telescoping technique and correlated them with the available clinical outcome. We found a good agreement between numerically predicted velocity changes and clinically observed treatment result, showing that computational fluid dynamics (CFD) simulations have a potential to be used in clinical practice to support planning of complex interventions.

## Methods

### Clinical case

**Fig. 1:**
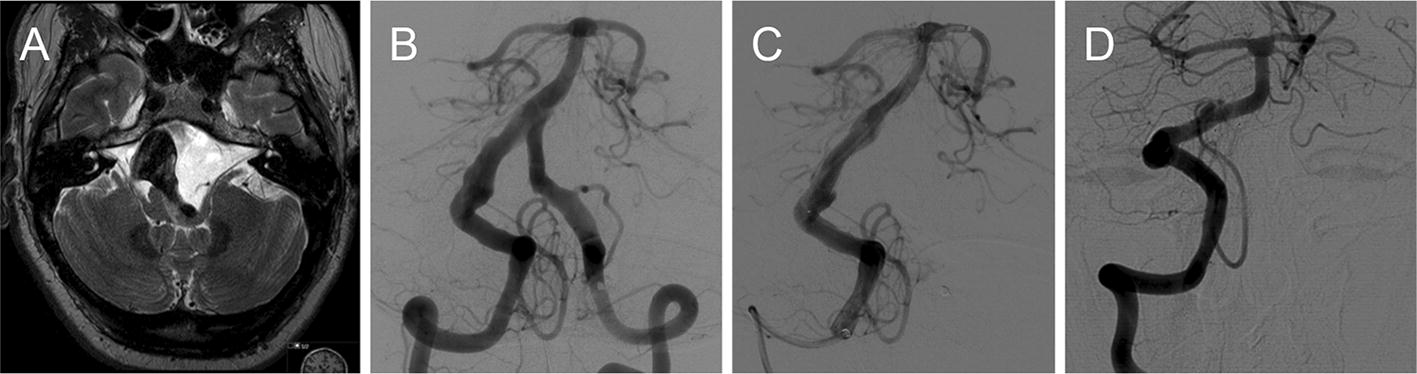
**A** T2-weighted MRI (magnetic resonance imaging) showing the large, partially thrombosed fusiform aneurysm of the basilar artery with mass effect and edema of the brainstem; **B** preinterventional DSA (digital subtraction angiography) run showing the partially thrombosed aneurysm as well as the dysplastic V4 segments of both vertebral arteries; **C** DSA run after placement of three flow-diverter stents in the basilar artery and right vertebral artery. Additionally the left vertebral artery was endovascularly occluded. **D** DSA control 1 year after the procedure shows a complete occlusion of the aneurysm with a sufficient restitution of the vessel anatomy of the treated V4 segment of the right vertebral artery

**Fig. 2:**
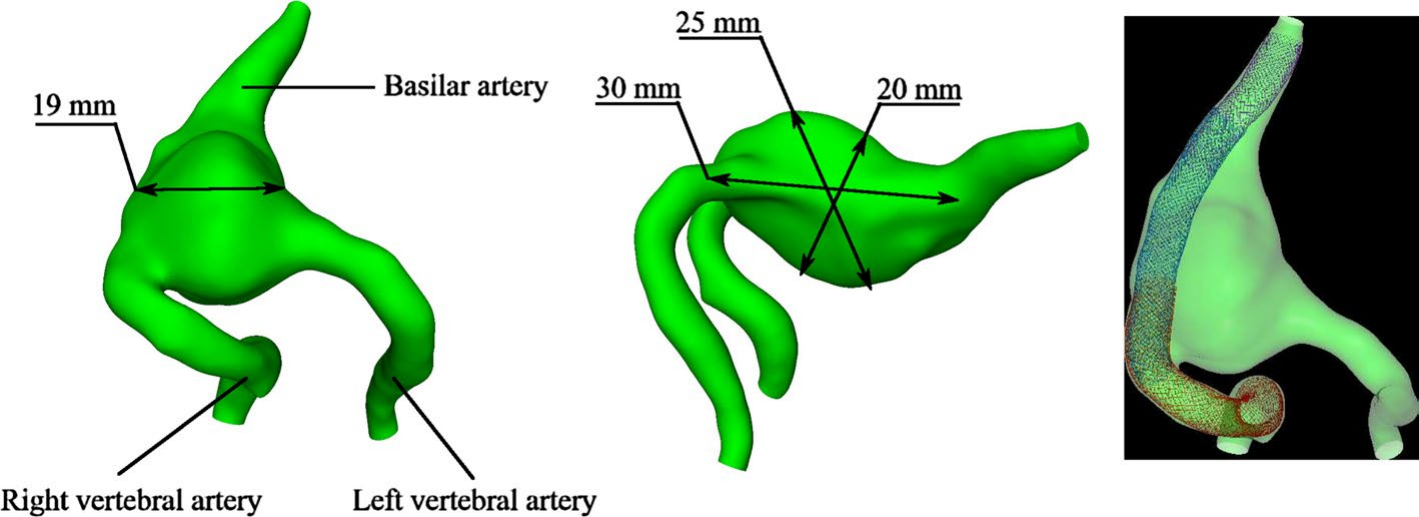
Giant fusiform aneurysm of the vertebrobasilar junction at different projections (at the left and at the middle); three flow-diverters deployed in series (at the right). Different colors differentiate between the devices

Patient-specific image data (Fig. 1) were acquired by 3D rotational angiography performed on a biplane Philips Allura Xper FD20 system (Philips Medical Systems B.V., Best, the Netherlands). The data were segmented with open-source software 3DSlicer 4.8.1 (http://www.slicer.org) and further post-processed with the Vascular Modeling Toolkit 1.3 (http://www.vmtk.org) [19]. Aneurysm shape and dimensions are presented in Fig. 2.

The site of the aneurysm is a special anatomical site in the human body, as it is the only region where two vessels (vertebral arteries) merge to become one vessel (basilar artery). This causes disturbed flow that may play a role in aneurysm development. From a clinical point of view, only one vessel is needed (and there are norm variants where only one vertebral artery is present). To reduce the probability of disturbed flow, it is a common clinical practice to occlude the smaller vertebral artery just before this merging point. Stenting of both arteries would result in the two stents touching each other in the central part of the basilar artery as they cannot be woven into each other. Such a scenario showed to result in major thromboembolic complications as it usually prevents a re-endothelialization of these touching stent parts.

Since the studied giant fusiform aneurysm could not be treated with a single flow-diverter stent, three flow-diverter SILK stents (Balt Extrusion, Montmorency, France) of 4.5 × 40, 5 × 40 and 5.5 × 40 mm, were deployed in series along the basilar and right vertebral arteries in a standard telescopic technique with an overlap of about 30%, i.e., at least 1 cm between the three single stents. In detail, first the distal stent was deployed using a conventional technique. However, after deployment, the central wire was not removed, but a microcatheter again advanced through the first flow-diverter stent, and a second flow-diverter stent was deployed in an overlapping manner, with an overlap of about 1 cm. This procedure was repeated again to cover the full length of the aneurysm. Additionally, the left vertebral artery was occluded endovascularly.

As our experiments were conducted retrospectively with permanently anonymized patient data, our local ethics committee deemed the study exempt from the requirement for approval.

### Virtual stenting

Geometrical models of the clinically used flow-diverter stents were reconstructed according to manufacturer specifications and deployed using a fast virtual stenting technique [20], which was previously validated on a set of real clinical cases [21, 22]. The deployment procedure started from computing a centerline from the right vertebral artery along the basilar artery. Then, the flow-diverter models were deployed one by one in series, starting from the largest one to the least. The overlapping between two neighboring stents was about 30% of the stent length. To estimate malapposition, for each overlapping region between two neighboring stents a set of five cross sections was used. The cross sections were equally distributed along the overlapping region. For each cross section, an area between the outer and inner stents was measured. Then the measured area was divided by the lumen area of the inner stent. The observed malapposition between *d* = 5.5 mm and *d* = 5 mm flow-diverter stents ranges between 10 and 12%. A range of 11 to 12% occurred between *d* = 5 mm and *d* = 4.5 mm flow-diverters. The result of the virtual deployment is presented in Fig. 2 (at the right). The final deployment was carefully inspected by three experienced neurointerventionalists and showed a sufficient agreement with the real deployment.

### Numerical simulations

Since patient-specific flow data were not available for the studied case, a realistic velocity curve was scaled to match an average flow rate of 100 ml/min, which is in physiological range of the vertebral arteries [23]. For the pretreatment setting, the flow rate in the left and right vertebral arteries was assumed to be equal. The maximum Reynolds numbers (*Re*) for the left and right vertebral arteries were 227 and 251, respectively; the average Re numbers were 151 and 160, respectively. A plug velocity profile was imposed at both inlets. Since inlet segments of the left and right vertebral arteries had sufficient lengths, realistic velocity profiles were developed in the aneurysm inflow zone. The free outflow condition was imposed at the outlet. The vessel wall was assumed rigid. Additionally, for the treated setting a zero inlet flow rate was imposed at the inlet of the left vertebral artery, since the left vertebral artery was occluded during the treatment.

Blood was considered as a Newtonian fluid with a dynamic viscosity $\mu$ of 3.5 mPas and density $\rho$ of 1050 kg/m^3^. A computational mesh was generated using snappyHexMesh tool from the OpenFOAM CFD Toolbox version 5.0 (OpenFOAM Foundation Ltd, London, UK). The mesh consisted primarily of hexahedral elements with a base size of 0.125 mm both for the untreated case and for the case with telescopic stenting. Additional refinement procedure was conducted to ensure a precise evaluation of the velocity gradients near the vessel wall and near the flow-diverters braiding [24]. To ensure grid independency, a mesh doubling test was carried out for both cases. The simulations were conducted with different base element sizes: 1 mm, 0.5 mm, 0.25 mm and 0.125 mm. The simulation results for the base element sizes of 0.25 and 0.125 mm differ only on about 1–2%. No significant change in the results was found, proving the numerical solution was accurate. The final mesh size was 5 million elements for the untreated case and 27 million elements for the case with flow-diverters.

Duration of the cardiac cycle, $T$ was set to 1 s. The Navier–Stokes equations for an incompressible fluid and continuity equation were solved using PIMPLE (merged PISO-SIMPLE) algorithm with OpenFOAM CFD Toolbox version 5.0. PIMPLE algorithm ensures numerical stability at higher time discretization steps and allows computation even in the cases where Courant number is greater than 1. A Gauss linear scheme was used for spatial discretization, whereas a Crank–Nicolson scheme was used for a temporal discretization. Transient CFD simulations before and after the treatment were performed under physiologically relevant pulsatile flow conditions. A time period of seven cardiac cycles was sufficient to reach a converged periodic solution with a time discretization step of 1 ms. The numerical results for the last (seventh) cardiac cycle were used for post-processing. An open-source package for scientific visualization ParaView 5.0.1 (Kitware, New York, USA) was used for visualization of the velocity field, whereas in-house developed software was employed for the calculation of hemodynamic parameters.

### Hemodynamic parameters

For correct comparison between the cases, only the aneurysm volume itself was considered excluding the flow-diverters. The following hemodynamic parameters were analyzed before and after the treatment.

A flow reduction $$R$$:

1 $$R = \frac{{U_{\text{avg}}^{\text{pre}} - U_{\text{avg}}^{\text{post}} }}{{U_{\text{avg}}^{\text{pre}} }} \cdot 100\% ,$$

where $U_{\text{avg}}^{\text{pre}}$ and $U_{\text{avg}}^{\text{post}}$ are space-averaged velocities in the aneurysm before and after the treatment, respectively.

Wall shear stress (WSS) distribution:

2 $$\overrightarrow {\text{WSS}} = \mu \frac{{{\text{d}}u}}{{{\text{d}}n}},$$

where $\frac{{{\text{d}}u}}{{{\text{d}}n}}$—velocity gradient along the normal to the vessel wall.

Since WSS changes during the cardiac cycle, the additional parameters were employed to characterize the shear stress acting on the vessel wall-TAWSS (time-averaged WSS) and TAWSSV (time-averaged WSS vector), which are defined as follows:

3 $${\text{TAWSS}} = \frac{1}{T}\mathop \smallint \limits_{0}^{T}\ \left| {\overrightarrow {\text{WSS}} } \right|{\text{d}}t,$$

4 $${\text{TAWSSV}} = \frac{1}{T}\left| {\mathop \smallint \limits_{0}^{T} \overrightarrow {\text{WSS}} \;{\text{d}}t} \right|.$$

TAWSS shows the average magnitude of WSS during the cardiac cycle, whereas TAWSSV characterizes the magnitude of the resulting shear stress vector.

Also, the oscillatory shear index (OSI) was used for evaluation of rate of change in shear stress vector:

5 $${\text{OSI}} = \frac{1}{2}\left\{ {\frac{{\left| {\mathop \smallint \nolimits_{0}^{T} \overrightarrow {\text{WSS}} \;{\text{d}}t} \right|}}{{\mathop \smallint \nolimits_{0}^{T} \left| {\overrightarrow {\text{WSS}} } \right|{\text{d}}t}}} \right\},$$

where OSI of 0.5 corresponds to completely oscillatory WSS, whereas OSI of 0 corresponds to unidirectional. Additionally, to characterize thrombogenic conditions in the aneurysm, the relative residence time (RRT) was calculated as follows [25, 26]:

6 $${\text{RRT}} = \frac{1}{\text{TAWSSV}}.$$

RRT represents a residence time of blood near the vessel wall.

## Results

**Fig. 3:**
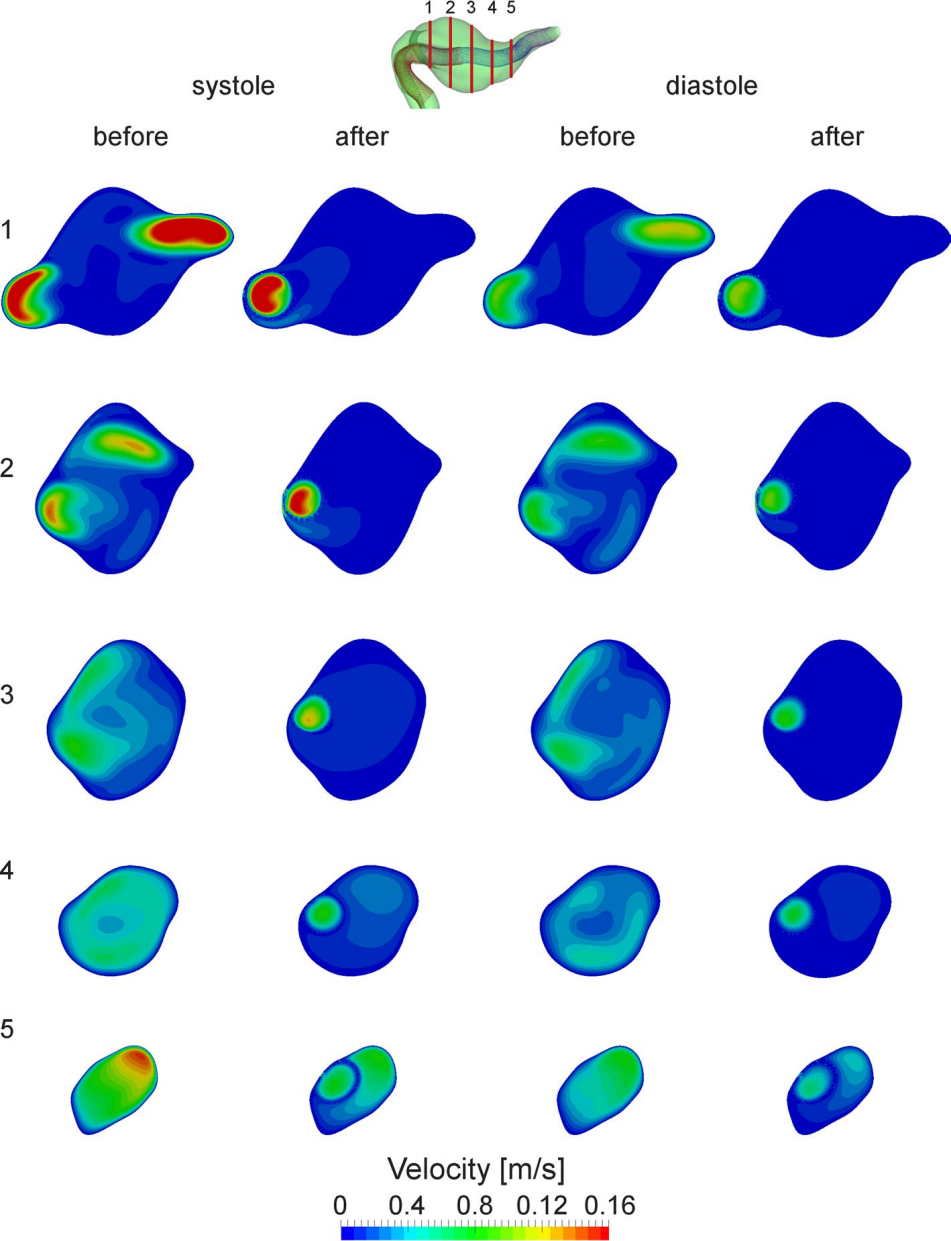
Velocity distribution at five aneurysm cross sections before and after the treatment during the systolic peak (*t*_*s*_ = 0.18 s) and diastolic end (*t*_*d*_ = 1 s)

**Fig. 4:**
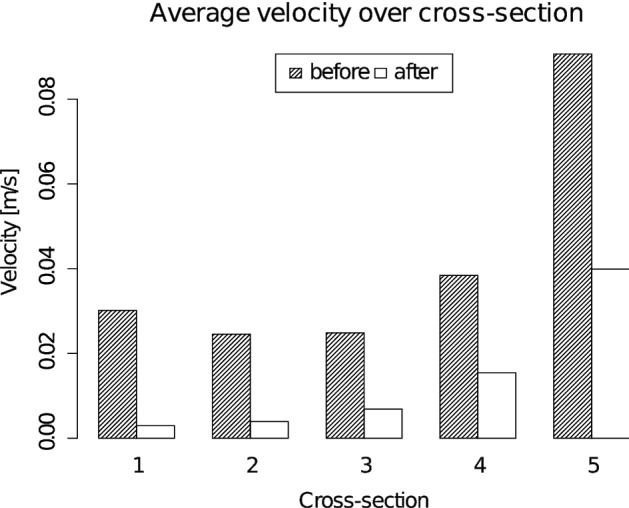
Space-averaged velocity over the cross sections during the systolic peak before and after the treatment

Figure 3 shows the comparative images for both the systolic peak and diastolic end. A set of five cross sections was used for qualitative comparison of velocity distribution in the fusiform aneurysm before (baseline) and after the treatment. The first one corresponds to the junction point between the left and right vertebral arteries, while the fifth belongs to basilar artery segment distal to the aneurysm. It should be noted that the second and fifth cross sections highlight the flow pattern in the overlapping regions between the first and second as well as the second and third flow-diverters, respectively. The baseline case is characterized by a complex flow structure with two high-velocity regions (from first to third cross sections), corresponding to inflow jets from the left and right vertebral arteries. In all considered cross sections, the high-velocity region is successfully jailed by the flow-diverters deployed in telescoping technique. For each overlapping region between two neighboring stents, a set of five cross sections was used. The cross sections were equally distributed along the overlapping region. For each cross section, only near-zero velocity values were observed in the tiny gap between the stents. Despite some malapposition between the overlapping flow-diverters (10–12%), the comparative images show clearly that there is no inflow into the aneurysm through the gaps in the telescopic construction. Only at the moment of systolic peak, a tiny amount of blood flows into the aneurysm between flow-diverters pores, which does not ultimately change the total high flow reduction in the aneurysm region (excluding the flow-diverter volume) observed at each cross section (see Fig. 4). Hence, at each aneurysm cross section the flow reduction during the systolic peak is higher than 55% with an average value of 72.4%.

For more information, regarding the flow reduction, the observed space-averaged velocities in the aneurysm region during the systolic peak and diastolic end for baseline and treatment case are summarized in Table 1.

**Table 1:** Velocity values at five aneurysm cross sections before and after the treatment

Cross-sectional number	1	2	3	4	5
Maximum velocity (baseline)	20.9	13.1	7.9	6.1	13.9
Maximum velocity (treated)	1.8	1.3	1.3	2.3	7.6
Maximum velocity reduction, %	91.5	89.8	83.4	62.3	44.8
Space-averaged velocity at systolic peak (baseline)	3.1	2.4	2.5	3.8	9.1
Space-averaged velocity at systolic peak (treated)	0.3	0.4	0.7	1.5	3.9
Space-averaged velocity reduction at systolic peak, %	90.2	84	72.3	59.8	55.9
Space-averaged velocity at diastolic end (baseline)	1.6	2.0	2.1	2.6	5.0
Space-averaged velocity at diastolic end (treated)	0.1	0.2	0.3	0.7	1.6
Space-averaged velocity reduction at diastolic end, %	91.9	92.2	86.2	76.7	67.9

All velocities are in cm/s

For both systolic peak and diastolic end, the intra-aneurysmal flow is substantially reduced by 56–92%. It should be noted that average velocity reduction for the diastolic end is higher than for the systolic peak, 83% against 72%, respectively. Moreover, the flow-diverter placement leads to vanishing of high-velocity zones in the aneurysm cavity, resulting in a reduction in maximum velocity values by 58% in average.

**Fig. 5:**
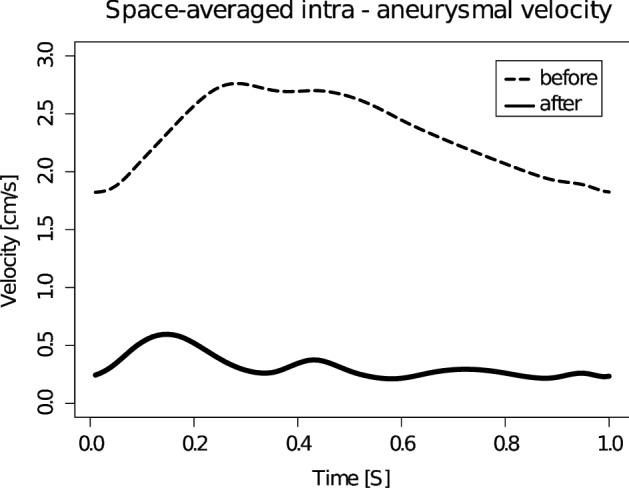
Space-averaged intra-aneurysmal velocity during the cardiac cycle before and after stenting

Analysis of space-averaged velocity in the aneurysm region, excluding the flow-diverter volume, revealed a major change in the trend for the treated case compared to the baseline (Fig. 5). The space-averaged velocity trend after the stenting mimics the inlet velocity curve with the peak value at 0.18 s, while the baseline demonstrates monotonous increase until the peak value at 0.25–0.45 s with a slight decrease until the diastolic end. Most probably, this is due to a good inlet pulse wave transmission along the flow-diverters, whereas for the baseline case the pulse wave is absorbed by the aneurysm walls. Nevertheless, the flow reduction is sufficient to produce thrombogenic conditions: The flow reduction $R$ = 86 ± 4.7% varies from the minimum of 74% to the maximum of 91% during the cardiac cycle.

**Fig. 6:**
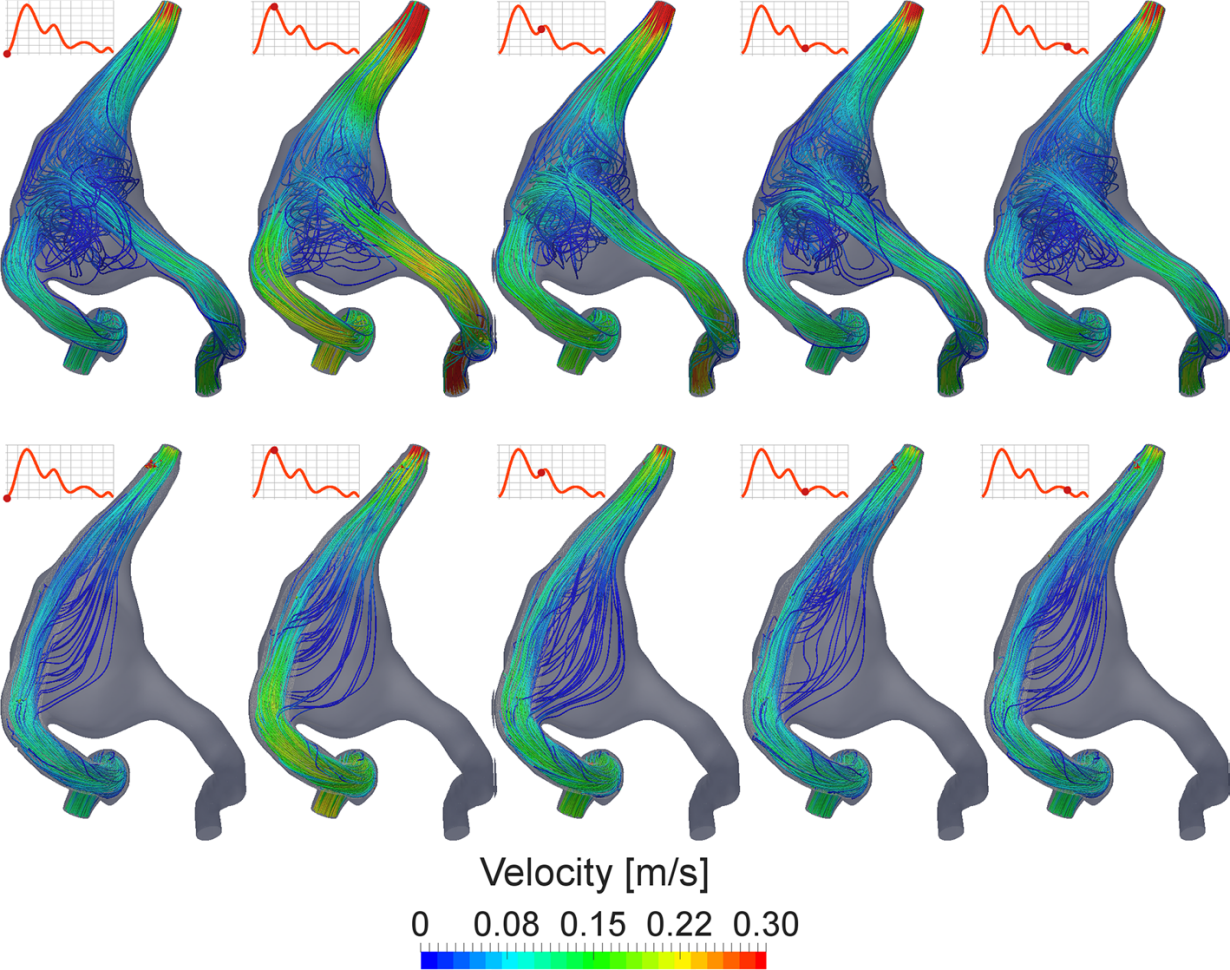
Intra-aneurysmal flow structure illustrated by streamlines before (first row) and after the telescopic stenting (second row) at five time points: *t* = 0, 0.2, 0.4, 0.6 and 0.8 s

The computed streamlines clearly demonstrate the major alteration of the flow pattern in the aneurysm after the treatment. The telescopic stenting and occlusion of the left vertebral artery allow to redirect the main inflow jet along the series of the flow-diverters during the cardiac cycle. To compare the flow structure before and after stenting, five time points were selected, i.e., 0, 0.2, 0.4, 0.6 and 0.8 s. The flow-diverting effect is clearly visible at all considered time points during the heart beat (Fig. 6). Moreover, the complex intra-aneurysmal flow structure with vortices, observed for the baseline case, disappeared after treatment, producing streamlines aligned to the main flow direction along the basilar artery.

**Fig. 7:**
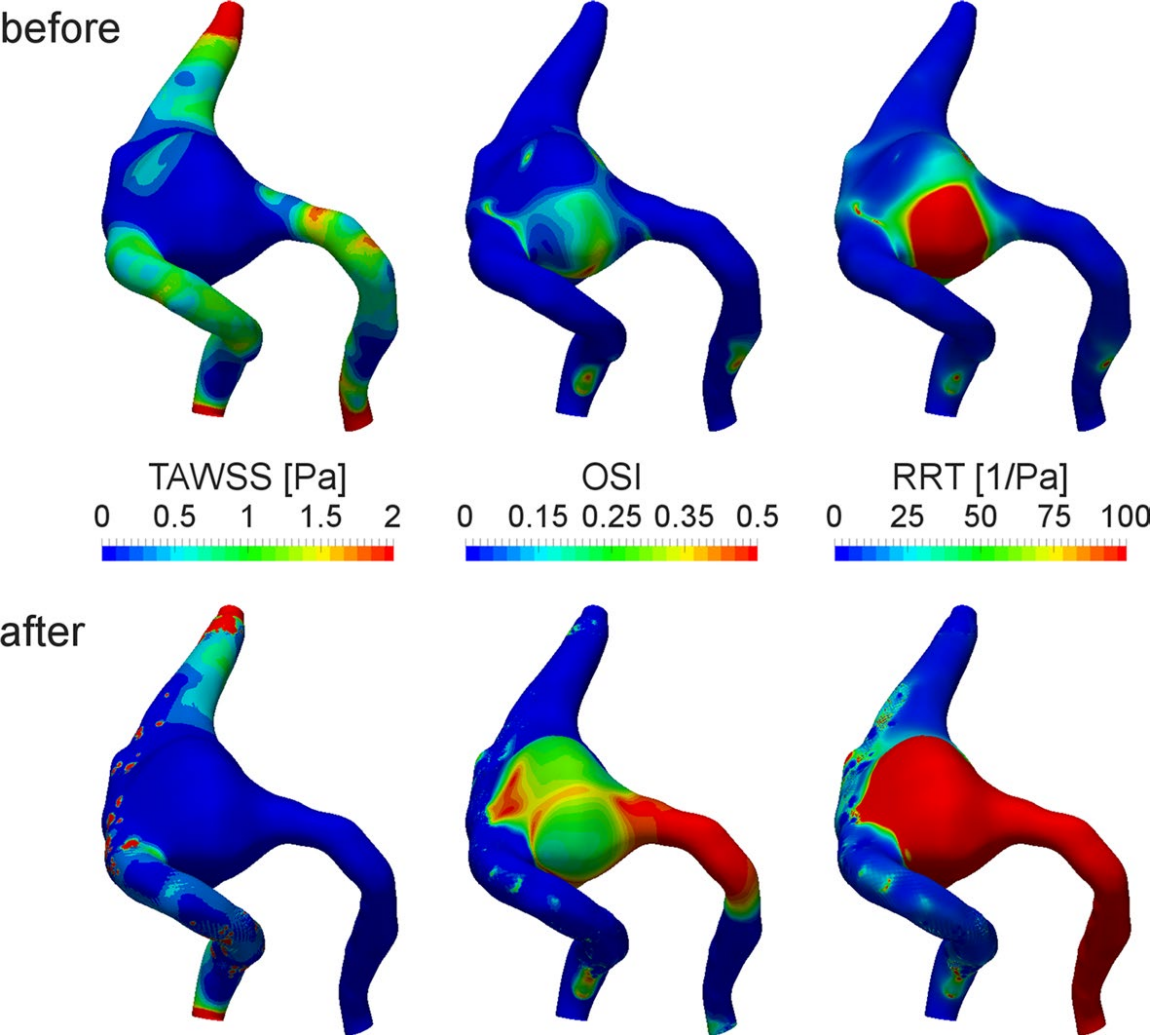
Distribution of hemodynamic parameters before and after the treatment in the fusiform aneurysm of the vertebrobasilar junction

Additionally, the simulated flow fields were post-processed to compute the hemodynamic parameters for quantitative comparison. As a result, the major changes after the telescopic stenting were revealed for distributions of the hemodynamic parameters (Fig. 7). TAWSS decreases at both the left (occluded) and right (stented) vertebral arteries, and a decrease by 85% is observed for TAWSS on the aneurysm dome itself. On the other hand, the flow alterations after the treatment produce the major elevation of OSI and change in its distribution pattern. The highest OSI values are observed, particularly, at the occluded left vertebral artery and at the aneurysm dome with the peak value of 0.45 and average value of 0.18, which are much higher than for the baseline case with 0.42 and 0.08 values, respectively. This demonstrates an increase in WSS vector fluctuations during the cardiac cycle at these specific regions. Moreover, a substantial elevation of RRT compared to baseline is found for both the occluded left vertebral artery and aneurysm dome (6.2 times), which indicates a flow stasis in the aneurysm and contributes to the formation of necessary thrombogenic conditions for the later aneurysm occlusion, which was observed during the follow-up after a 1 year.

## Discussion

Several multicenter studies have reported a success in 80–90% of cases treated with flow-diverters, especially for saccular intracranial aneurysms of the anterior circulation [27, 28]. Despite the recent achievements for anterior circulation aneurysms, the treatment of fusiform aneurysms of the posterior circulation with flow-diverters is quite challenging, due to high risk of post-treatment complications and relatively low rate of clinically successful cases [29]. Moreover, the prediction of post-treatment flow alterations is still challenging, especially for complex aneurysms treated with multiple flow-diverters. Therefore, a comprehensive analysis of their application should be conducted before flow-diverter stents can become clinical routine for the treatment of posterior circulation aneurysms.

The effectiveness of flow-diverters for posterior circulation aneurysms was investigated by a number of studies [17]. In the largest studied cohort today, including 131 cerebral aneurysms of the posterior circulation, Griessenauer et al. [1] evaluated safety and efficacy of the treatment with flow-diverters individually for each aneurysm type. They found no clinical predictors of occlusion (such as age, sex, aneurysm size and location) for posterior fusiform aneurysms, making rupture risk evaluation and treatment prognosis challenging. In such a case, hemodynamic parameters should be considered as well to provide an insight into the flow field in the aneurysm region. This is especially important in the case of complex stenting, where post-treatment flow alterations are difficult to predict. Hence, Lv et al. [30] have recently studied hemodynamics in vertebral artery fusiform aneurysms treated with three overlapping self-expanding Enterprise stents and found favorable changes in hemodynamic parameters, leading to thrombosis formation after the stenting. However, the effect of telescopic flow-diverter placement in a giant fusiform aneurysm of the vertebrobasilar junction on blood flow was not studied yet, which is an objective of the present study.

In this study, we analyzed hemodynamic performance of multiple flow-diverters deployed in series in a giant fusiform aneurysm of the vertebrobasilar junction and correlated flow changes with the known clinical outcome. The main analyzed hemodynamic parameters like velocity and WSS distribution as well as WSS-derived parameters such as OSI, TAWSS, TAWSSV and RRT were evaluated before and after the treatment. We observed a major flow reduction by 78% in the aneurysm as well as subtle changes in the flow pattern, WSS distribution and other hemodynamic parameters after the treatment. The observed flow reduction and elevated RRT indicate that red blood cells and platelets stay longer in the sac of the aneurysm. This induced a flow stasis and increased the residence time in the aneurysm region, resulting in thrombogenic flow conditions. The results of the simulation correlate well with the known successful outcome during the follow-up period for the studied clinical case. Further, we found telescopic stenting to be effective for treating the giant fusiform aneurysm of the basilar artery in the considered case with no inflow to the aneurysm from regions of contact between the neighboring stents. The results of our study correlate with the recent study by Ouared et al. [31], proposing that a flow reduction of at least one-third is sufficient for sidewall aneurysm occlusion after flow-diverter placement. In our case, we observed that a flow reduction of about two-third was sufficient for the fusiform aneurysm occlusion.

Since fusiform aneurysms are associated with degenerative processes within the vessel wall, changes in WSS distribution are of particular interest [32]. It is well known that magnitude and direction of WSS correlate with the regulation of biochemical processes in the endothelium cells, which form an inner layer of the vessel wall, and directly influenced by the moving blood. Both low and high WSS values could trigger degenerative changes in endothelial cells and cause the disorder of cell matrix integrity [33]. In our study, we observed the substantial reduction in TAWSS in both the aneurysm sac and (occluded and not occluded) vertebral arteries after the stent deployment, which agrees well with the study by Xiang et al. [34], demonstrating the elimination of high WSS region from the aneurysm dome after flow-diverter placement. Additionally, we revealed the elevation of OSI in both the aneurysm and occluded vertebral artery, which could be a result of low-velocity fluctuations in these regions. Our results are in correlation with another study by Xiang et al. [35], proposing that TAWSS and OSI are the only independently significant variables which are discriminate rupture status of intracranial aneurysms. The major increase in RRT in both the aneurysm and the occluded vertebral artery clearly demonstrates the desired flow-diverting effect of the stenting, producing a flow stasis and thrombogenic flow conditions, which ensure effectiveness of the treatment. This agrees well with the results of the study by Li et al. [16] reporting that a significant increase in RRT was observed in successfully treated fusiform aneurysms of the posterior circulation.

Such numerical simulations of blood flow could be used in clinical practice to plan complex stent deployment and estimation of possible risk factors associated with a deployment in fusiform aneurysms, as well as for large aneurysms, where a complex treatment with multiple stents in a telescopic technique is necessary. On the first step, pretreatment hemodynamics conditions (baseline) in a posterior aneurysm should be determined by evaluating the main hemodynamic parameters in the aneurysm sac [36]. After that, each treatment option (flow-diverter model, number of stents, percent overlap of stents and others) could be evaluated by numerical simulations accompanied by fast virtual stenting and compared with the baseline to find out the configuration leading to favorable changes in post-treatment hemodynamics (maximum flow reduction, subtle increase in RRT, etc.).

The presented study has some limitations which could influence the results. First, only the general inlet velocity waveform, plug velocity profile at the inlet and constant pressure level at the outlets were used, since patient-specific data were not available for the considered clinical case. Second, the applied fast virtual stenting technique could produce slight differences for the telescopic stenting compared to real deployment scenario. Third, the assumption of a Newtonian fluid might lead to overestimation of velocity magnitude and other velocity-derived parameters like WSS, TAWSS, etc. in the aneurysm sac, where low-shear-rate regions are observed [37, 38]. Also, deformation of stent was not considered. Additionally, neglecting elastic properties of the aneurysm wall could also result in overestimation of the hemodynamic parameters considered in the study [39]. Finally, an unsuccessful case was not modeled and multiple different cases of complex stent deployment were not considered, limiting the generalizability of obtained results to different deployment techniques that are associated with successful versus adverse outcomes. Future work will address the listed limitations.

## Conclusions

Numerical simulations showed telescopic stenting to be effective for treating the giant fusiform aneurysm of the basilar artery with no inflow to the aneurysm from regions of contact between the neighboring stents. Observed major flow reduction by 78% and subtle changes in the hemodynamic parameters created thrombogenic flow conditions in the aneurysm. We found a correlation between the numerically predicted flow alterations and the available treatment outcome showing a potential of image-based simulations to be used in clinical practice for the treatment planning and estimation of possible risk factors associated with a complex stent deployment in fusiform aneurysms of the posterior circulation.

## Data Availability

Data can be shared upon request.

## Abbreviations

CFD: computational fluid dynamics
DSA: digital subtraction angiography
MRI: magnetic resonance imaging
OSI: oscillatory shear index
RRT: relative residence time
TAWSS: time-averaged WSS
TAWSSV: time-averaged WSS vector
WSS: wall shear stress
